# Delayed Maturation of Oligodendrocyte Progenitors by Microgravity: Implications for Multiple Sclerosis and Space Flight

**DOI:** 10.3390/life12060797

**Published:** 2022-05-27

**Authors:** Victoria Tran, Nicholas Carpo, Sophia Shaka, Joile Zamudio, Sungshin Choi, Carlos Cepeda, Araceli Espinosa-Jeffrey

**Affiliations:** 1 Department of Psychiatry, Semel Institute for Neuroscience and Human Behavior, The University of California Los Angeles, Los Angeles, CA 90095, USA; victoriakimtran@gmail.com (V.T.); npgcarpo@gmail.com (N.C.); shaka@g.ucla.edu (S.S.); joilezmd@gmail.com (J.Z.); ccepeda@ucla.edu (C.C.); 2 KBR, NASA Ames Research Center, Moffett Field, CA 94035, USA; sungshin.y.choi@nasa.gov

**Keywords:** microgravity, oligodendrocyte progenitors, incomplete cytokinesis, transferrin, human neural stem cells, cell proliferation, space flight

## Abstract

In previous studies, we examined the effects of space microgravity on human neural stem cells. To date, there are no studies on a different type of cell that is critical for myelination and electrical signals transmission, oligodendrocyte progenitors (OLPs). The purpose of the present study was to examine the behavior of space-flown OLPs (SPC-OLPs) as they were adapting to Earth’s gravity. We found that SPC-OLPs survived, and most of them proliferated normally. Nonetheless, some of them displayed incomplete cytokinesis. Both morphological and ontogenetic analyses showed that they remained healthy and expressed the immature OLP markers Sox2, PDGFR-α, and transferrin (Tf) after space flight, which confirmed that SPC-OLPs displayed a more immature phenotype than their ground control (GC) counterparts. In contrast, GC OLPs expressed markers that usually appear later (GPDH, O4, and ferritin), indicating a delay in SPC-OLPs’ development. These cells remained immature even after treatment with culture media designed to support oligodendrocyte (OL) maturation. The most remarkable and surprising finding was that the iron carrier glycoprotein Tf, previously described as an early marker for OLPs, was expressed ectopically in the nucleus of all SPC-OLPs. In contrast, their GC counterparts expressed it exclusively in the cytoplasm, as previously described. In addition, analysis of the secretome demonstrated that SPC-OLPs contained 3.5 times more Tf than that of GC cells, indicating that Tf is gravitationally regulated, opening two main fields of study to understand the upregulation of the Tf gene and secretion of the protein that keep OLPs at a progenitor stage rather than moving forward to more mature phenotypes. Alternatively, because Tf is an autocrine and paracrine factor in the central nervous system (CNS), in the absence of neurons, it accumulated in the secretome collected after space flight. We conclude that microgravity is becoming a novel platform to study why in some myelin disorders OLPs are present but do not mature.

## 1. Introduction

Nerve fibers that are wrapped in myelin can transmit their electric impulses faster than non-myelinated axons. Myelin makes saltatory conduction in nerve fibers possible through the nodes of Ranvier. Central nervous system (CNS) myelin disorders continue to be a major public health challenge due to the lack of adequate therapies. Two of the most notable conditions are brain white matter injury (WMI) found in premature infants and multiple sclerosis (MS) in adults [1] Oligodendrocytes (OLs) derive from oligodendrocyte progenitors (OLPs) and are the cells that synthesize, build, and maintain healthy myelin throughout life [2]. They are also the cells that are required for remyelination after injury or disease. Moreover, it has been reported that recapitulation of the same mechanisms occurring during development is necessary for remyelination [3,4,5,6]. Nonetheless, in most myelin disorders, the problem is not a lack of OLs but that the OLPs present at and around the lesion fail to mature [7].

We have established a number of chemically defined media for neural cells and, in particular, for OLs, starting in 1988 with the first medium for OLs [8]. Each one considers the specific nutrients, additives, and time windows when the additives should be provided to either make the cells remain at a specific developmental stage, i.e., progenitors (Espinosa et al., 1988), or move forward in the lineage. The most recent culture system aims at instructing human induced pluripotent stem cells (hiPS) to become mature, functional OLs [9]. We started with embryoid bodies (EBs) and thereafter directed and instructed their commitment to the neural and subsequent OLs phenotype. Once commitment of hiPS to the OLs stage was reached, the steps of lineage progression were bolstered by the culture systems we have already described [9]. Besides inducing and maintaining a specific phenotype, the importance of this culture system resides in the fact that we achieved phenotype commitment of hiPS to OLs without using gene transfer [10,11,12] or undefined substrates present in other methods, such as a different cell used as a substrate or a different cell-line-derived conditioned medium. Moreover, our system does not use animal serum or products.

With the advent of human induced pluripotent stem cells (hiPS) in 2006, for the first time, the possibility to make cells for transplantation where the donor would also be the recipient was a promise to all fields of regenerative medicine. With that in mind, we developed a culture system that allowed for the derivation of human oligodendrocytes (OLs) from (hiPS) and their further maturation. The use of very precise amounts of fresh additives and factors also contributes to the shorter periods of time necessary to obtain hiPS-derived OLs [13,14]. The considerable shortening of the time necessary to derive OLPs was the concomitant fate restriction and lineage specification of hiPS towards the neutral and OL phenotypes. The OLPs generated are viral- and integration-free and therefore very suitable for cell replacement therapies.

Reports in the literature have shown that cells respond differently to microgravity depending on the cell type: it induces apoptosis, alters the cytoskeleton, and affects signal transduction, differentiation, proliferation, migration, and adhesion. Yet, only a few aspects on the sensitivity of neural stem cells to weightlessness (0 G) have been examined [15]. In embryonic stem cells (ESCs), microgravity reduces the differentiation and regenerative abilities, preserving them in a “stemness” state [16]. This could lead to reduced or impaired regeneration of tissues in space in diseases and upon trauma preventing repair and regain of function. Interestingly, another study reported arrested proliferation of NSCs when in simulated microgravity (sim-µG) but not permanently, as NSCs recovered their proliferative capacity when they were returned to Earth’s gravity [17]. We have previously reported that sim-µG increased the proliferation of rodent and human OLPs with the concomitant shortening of the cell cycle [18]. We have also reported that sim-µG enhances OLPs’ mitochondrial function and lipid metabolism [18] and that NSCs proliferate more while in space and after space flight [19,20]. These findings led us to hypothesize that space microgravity would produce similar results with regard to OLPs. This is a subject of utmost importance, considering that space flight causes intracranial hypertension, bulging of the optic nerve, and derived disturbances, such as glaucoma conditions, that do not normalize upon return to Earth [21]. 

To start unraveling the mechanisms involved in this phenomenon, we prepared hiPS-derived OLPs and sent them to space onboard SpaceX-21. Using our OLPs culture system, we examined their behavior post-flight as they were adapting to Earth’s gravity using time-lapse microscopy. We also examined their ability to express developmental markers as they progressed from OLPs towards mature Ols. We found that OLPs survived and adapted well to Earth’s gravity after space flight. Nonetheless, based on morphology and immunocytochemistry, they appeared to return in a “younger-like” stage than when they were seeded for space flight. This was corroborated by the persistent expression of OLP-specific markers. 

To make OLPs flown to space (SPC-OLPs) mature upon return to Earth, we exposed them to the appropriate medium to make the cells move forward to their next developmental stage; however, this was unsuccessful. Ground control (GC) OLs did proceed to a more mature phenotype. Therefore, here, we report for the first time the modulation of the expression of stage-specific developmental OLP markers and, in particular, Tf expression in the nucleus after space flight. To our knowledge, this is the first paper describing the effects of space microgravity on human OLPs. This work is relevant to humanity because it confirms that microgravity serves as a novel platform to study why in myelin disorders like MS or after injury, OLPs are present but do not mature. Moreover, this approach can be used to develop new therapeutic and preventive strategies to be used on Earth before space flight journeys and as countermeasures for astronauts in long-term space missions. It is therefore critical to understand the impact of space microgravity on OLPs’ development.

## 2. Materials and Methods

### 2.1. Cells and Culture System

**Figure 1:**
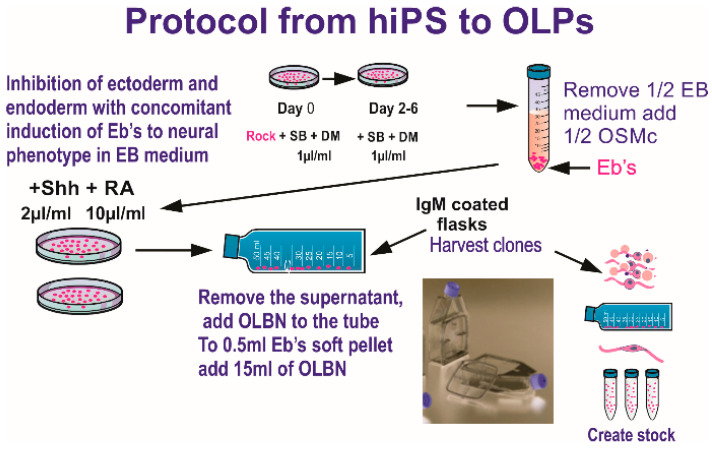
Ectoderm selection was performed by exposing hiPS to SB431542 (SB) and dorsomorphin (DM) to instruct ectoderm formation preferentially, while inhibiting endodermal and mesodermal commitment. Once specified, a stock of hiPS-derived OLPs was created for all pre-flight and post- flight experiments in order to keep passage number low (18 passages maximum). Details for the procedure described here have been published [9]). This medium is designed to induce OLP phenotype and maintain these progenitors proliferating. OLPs flew to space in this culture medium. The composition of “OSM” (oligodendrocyte specification medium) is shown in Table 1.

Prior to the space flight, a homogeneous population of NSCs was obtained from hiPS. The original hiPS cell line “CS83iCTR-33nxx” (fibroblasts) was “reprogrammed” and provided to us by Cedars-Sinai Medical Center via a material transfer agreement. These cells were converted to “OLPs” using the protocol shown in Figure 1.

Dissolve the listed components in 500 mL of D-MEM/F12 medium (High glucose) to make OSMb (basal). To prepare OSM complete (OSMc), add 10 µL of both N2 and B-27 with retinoic acid to OSMb. To prepare “welcoming medium”, we used OSMb + IGF-1 (final concentration of 88–100 ng/mL, freshly prepared) without B-27 or N2 and allowed to recover for six hours in the incubator before placing them in the imaging system (Supplemental Table S1).

**Figure 2:**
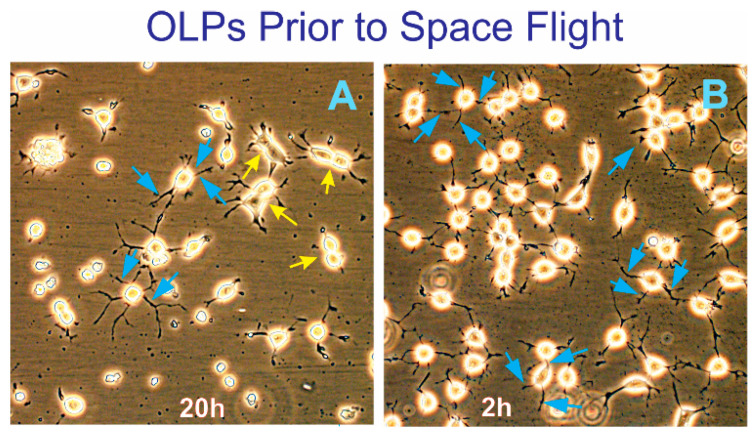
(**A**) View of OLPs before the space flight. View of hiPS-derived OLPs 20 h after being thawed. Most cells displayed three or four cell processes (blue arrows). Some were actively dividing (yellow arrows). (**B**) View of OLPs just prior to harvesting and plating in the flying hardware. The initial seeding cell density was 0.7 × 10^6^ OLPs per well.

Two years before the space flight, the cell line was tested to ascertain the conditions, such as cell numbers, survival, OLPs’ proliferation, hardware material compatibility, starting number of cells, and so on. To integrate the space flight, frozen hiPS-OLP cultures passage 10 were started at Kennedy Space Center (KSC), and a large stock was created for both the space flight and ground control experiments. OLPs were maintained at 36.8 °C and 4.5% CO_2_ and fed every other day (Figure 2). 

### 2.2. Hardware and Space Flight

**Figure 3:**
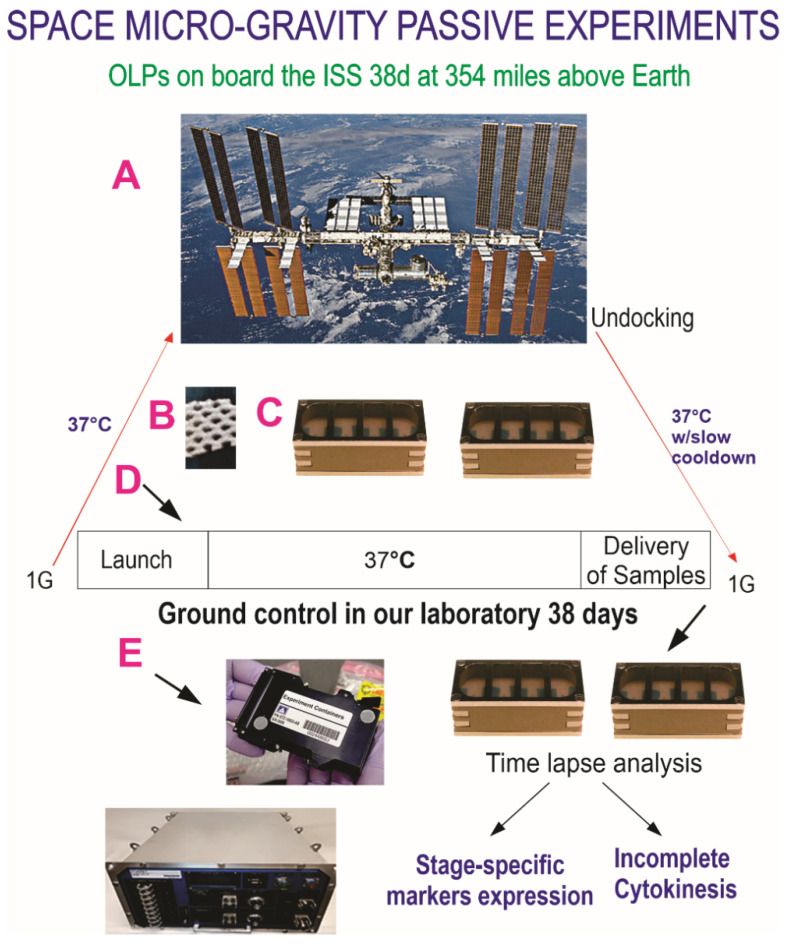
Synopsis of Passive experiments. OLPs as part of the Bioscience-4 Space Biology NASA experiment, flown to the ISS on board SpaceX-21 (**A**). It was launched on 6 December 2020 and landed (splash-down) on 13 January 2021. The cells remained in microgravity for approximately 38 days. Its orbit height was 254 miles; its speed on orbit was 4.76 miles/s; and maximal speed reached 17,400 mph. On Earth, humans are exposed to 3 to 4 millisieverts (mSv) of radiation from natural sources, per year, mainly from cosmic rays that make it through the atmosphere. On the International Space Station, astronauts receive about 150 mGV per six months. For the current mission, the average daily total radiation dose was 0.425 (mGv) on board the space station. This experiment is called “passive” because it was designed to mimic the trajectory astronauts’ brains undergo during space flight (i.e., launch, stay in space, and splash down when returning to Earth) without manipulation or medium change. (**B**) Example of mesh without cells. OLPs were seeded on the floating mesh carriers. (**C**) View of the 8-well Petri dishes used for the passive experiments on Earth. (**D**) Timeline and conditions of the automation and experiment while in space. At T + 2, the medium from the first tank was sent to the cell chamber, and it remained in contact with OLPs for 26 days (T + 28). At this time, the medium was removed from the cell chamber, and the medium from the second tank was sent to the cell chamber. (**E**) View of the Automated Type IV hardware used in our laboratory for GC cells. The same number of chambers was flown to space or stayed on Earth in our laboratory as ground control. All cells were maintained at 37 °C. Ground control cells were also seeded in these containers and maintained at the same temperature and conditions in our laboratory; the only difference was gravity vs. microgravity. Upon splash-down, they were transferred from Kennedy Space Center to World Carrier and brought to our laboratory (UCLA) at 37 °C (Figure modified from [20]).

The BioScience-4 mission launched onboard the Space-X 21 Dragon capsule on 6 December 2020. This is the first study to investigate the proliferation of human normal hiPS-derived OLPs in space microgravity. For space flight, OLPs were seeded onto passive 8-well Petri dishes from Airbus-Kiwi (Friedrichshafen, DE) on floating mesh carriers 2 mm × 2 mm to which cells adhered firmly; this feature was necessary to ensure that cells would not detach and die during launching or while returning to Earth. OLPs were flown to the International Space Station (ISS) and installed in the STAaRS F-1 Space Technology and Advanced Research System Experiment Facility-1 (STAaRS F-1) at 37 °C. Cells remained onboard the ISS for 39 days and 9 h and then returned to Earth (Figure 3). The activity schedule is shown in Table 2.

### 2.3. Time-Lapse of SPC-OLPs Behavior

Upon arrival, cells were seeded onto flasks or flaskettes and then were harvested and resuspended in a culture medium designed to allow them to move forward in the lineage. This culture medium was made with OSMb + IGF-1 (final concentration of 88–100 ng/mL, freshly prepared) without B-27 or N2 and allowed to recover for six hours in the incubator before placing them in the imaging system. For details on the functioning of this hardware, please see information from Yuri https://www.yurigravity.com/ accessed on 1 February 2022. We used the Zeiss Axio Observer 7 fully motorized inverted research microscope with the Zeiss Axiocam 506 monochrome camera with Zeiss ZEN software and definite focus equipped with the full Incubation XL chamber (Zeiss) for temperature and CO_2_ control with motorized scanning stage. 

### 2.4. Immunofluorescence 

Cells were examined for the expression of OLP markers such as Sox2, which is essential for OLPs’ proliferation during development [22] and sustains their recruitment during demyelination [23]. The iron carrier glycoprotein transferrin (Tf) is one of the earliest markers for OLPs and remains as a marker of two subpopulations of OLs in mature OLs in culture and in adulthood in vivo [24]. Platelet-derived growth factor receptor (PDGFR-α antibody, Cell Signaling), Olig2, and the Nkx2.2 homeodomain transcription factor govern OLs’ specification during development. Moreover, during remyelination, a transient upregulation of Nkx2.2 in OLPs has been reported [25]. Thus, Nkx2.2 determines the timing for OLs’ differentiation [26], and its dysregulation prevents OLs’ formation [27]. We also used the OL-specific enzyme glycerol phosphate dehydrogenase (GPDH) a mouse monoclonal antibody made in our laboratory, as it also labels OLPs later than Tf [28]. The anti-TF monoclonal antibody was (OTI5G2 TrueMABTA500848, Thermo Fisher; anti-SOX2 antibody ab97959; PE Anti-Hsp90 beta antibody ab11564).

Three secondary antibodies were used to visualize the markers mentioned above: anti-rabbit Texas red, 1:800 dilution; anti-mouse IgG Alexa Fluor 488, 1:1000 dilution; and anti-mouse IgM Alexa Fluor 633, all from ABCAM, 1:1000 dilution. Cells were incubated in this cocktail for 1 h at room temperature, washed with Tris-buffered saline (TBS), and mounted. The samples were imaged with the LSM 800 confocal microscope (Zeiss, Jena, Germany) and analyzed with the Zen software (Zeiss). 

### 2.5. Proteomics and Secretome

#### 2.5.1. Synopsis of the Space Flight for the Active Experiment Using Automated Type IV Units

**Figure 4:**
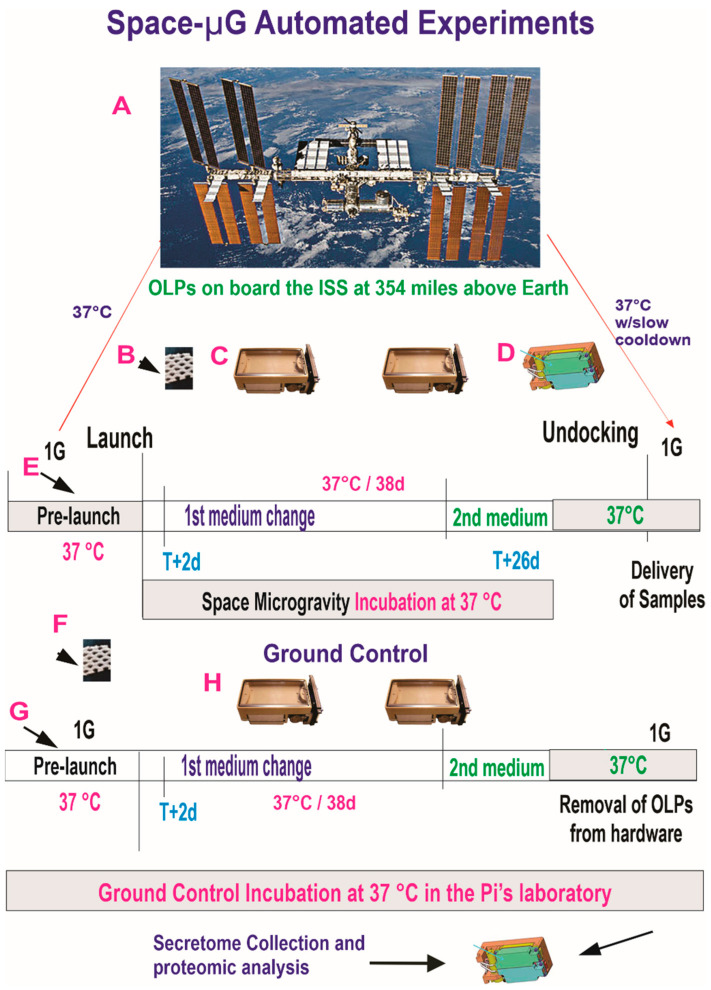
Synopsis of the automated experiments. OLPs, as part of the Bioscience-4 Space Biology NASA experiment, flew to the ISS on board SpaceX-21. The BioSci-4 experiment was launched on 6 December 2020 and landed (splash-down) on 13 January 2021. Therefore, the cells remained in microgravity for approximately 38 days. This experiment is called “active” because it used automated units to perform cell culture medium changes on demand. It was designed to collect the conditioned medium (secretome) after OLPs reached space and before unberth to capture the molecules secreted solely while cells were onboard the ISS (**A**). (**B**) View of the mesh carrier (2 mm × 2 mm). (**C**) Automated Type IV hardware used in our laboratory for GC cells. (**D**) View of the two tanks located underneath the cell container. (**E**) The timeline depicting the handover moment and pre-launch which lasted 48 h. (**F**) View of a mesh cell-carrier. (**G**) Timeline for events pre-flight on board the ISS and prior to unberth and conditions at which the cells travelled. The travel culture medium was removed at T + 2 and fresh medium was left with the cells for 26 d at which time the second culture medium was added to the cultures. The secretome produced while in space was contained in tank 2. OLPs travelled back from space in the second medium. The same number of chambers were flown to space or stayed on Earth in our laboratory as ground control. All cells were maintained at 37 °C. Ground control OLPs were also seeded in these containers and maintained at the same temperature and conditions in our laboratory; the only difference was Earth’s gravity vs. space microgravity. Upon splash-down, they were transferred from Kennedy Space Center to World Carrier and brought to our laboratory (UCLA) at 37 °C (Figure modified from [20]). (**F**) GC OLPs were also seeded on mesh carriers. (**H**) Two Type IV units were used for GC. All units travelled back from space at 37 °C with slow cool down. Upon arrival, cells were collected to seed them onto flaskettes for microscopy, and the secretome was also collected and stored at −80 °C.

The culture medium that fed the cells during space flight was recovered from the hardware separately (medium from the cell chamber) Figure 4, and from each tank was placed in separate numbered tubes with addition of proteases inhibitor cocktail and saved frozen at −80 °C. This medium is commonly known as the conditioned medium. For the purpose of this manuscript, we refer to this conditioned medium as “secretome”.

#### 2.5.2. Preparation of Samples 2-D DIGE 

The conditioned medium samples were thawed and vortexed for 20 s. The samples were spun at 4 °C and 14,000 rpm for 30 min. The supernatant was collected from the sample. For these samples, serum Albumin and IgG were removed using Thermo Scientific Albumin/IgG Removal Kit. Next, the depleted serum samples were concentrated and exchanged into 2-D Lysis buffer (7 M urea, 2 M thiourea, 4% CHAPS, and 30 mM Tris-HCl, pH 8.5). Protein concentration was measured in all samples using Bio-Rad protein assay method.

The reaction was stopped by adding 1.0 µL of 10 mM Lysine to each sample and incubating in the dark on ice for an additional 15 min. The labeled samples were then mixed. The 2X 2-D Sample buffer (8 M urea, 4% CHAPS, 20 mg/mL DTT, 2% pharmalytes, and trace amounts of bromophenol blue), 100 µL destreak solution, and Rehydration buffer (7 M urea, 2 M thiourea, 4% CHAPS, 20 mg/mL DTT, 1% pharmalytes, and trace amounts of bromophenol blue) were added to the labeling mix to make the total volume of 250 µL. We mixed well and spun before loading the labeled samples into the strip holder.

#### 2.5.3. IEF and SDS-PAGE

After loading the labeled samples, IEF (pH 3–10 Non-Linear) was run following the protocol provided by GE Healthcare. Upon finishing the IEF, the IPG strips were incubated in the freshly made equilibration buffer-1 (50 mM Tris-HCl, pH 8.8, containing 6 M urea, 30% glycerol, 2% SDS, 10 mg/mL DTT, and trace amounts of bromophenol blue) for 15 min with light shaking. Then, the strips were rinsed in the freshly made equilibration buffer-2 (50 mM Tris-HCl, pH 8.8, containing 6 M urea, 30% glycerol, 2% SDS, trace amounts of bromophenol blue, and 45 mg/mL Iodoacetamide) for 10 min with gentle shaking. Next, the IPG strips were rinsed in the SDS-gel running buffer before transferring into 13.5% SDS-gels. The SDS-gels were run at 15 °C until the dye front escaped out of the gels.

#### 2.5.4. Image Scan and Data Analysis

Immediately following the SDS-PAGE, gel images were scanned using Typhoon TRIO (GE Healthcare. The scanned images were then prepared for analysis utilizing Image Quant software (version 6.0, GE Healthcare). Additionally, an in-gel analysis was performed using DeCyder software (version 5.0, GE Healthcare). The fold change of the protein expression levels was obtained from in-gel DeCyder analysis.

#### 2.5.5. Spot Picking and Trypsin Digestion

Ettan Spot Picker (Amersham BioSciences) was utilized to pick spots of interest. The spots were determined by the in-gel analysis and spot picking function by DeCyder software. The gel spots were washed and then digested in-gel with modified porcine trypsin protease (Trypsin Gold, Promega). Zip-tip C18 (Millipore) was used to desalt the digested tryptic peptides. Peptides were eluted from the Zip-tip with 0.5 μL of matrix solution (α-cyano-4-hydroxycinnamic acid (5 mg/mL in 50% acetonitrile, 0.1% trifluoroacetic acid, 25 mM ammonium bicarbonate)) and spotted on the AB SCIEX MALDI plate (Opti-TOFTM 384 Well Insert).

#### 2.5.6. Mass Spectrometry

MALDI-TOF MS and TOF/TOF tandem MS/MS were performed on an AB SCIEX TOF/TOF™ 5800 System (AB SCIEX, Framingham, MA, USA). MALDI-TOF mass spectra were obtained in reflectron positive ion mode, with an average of 4000 laser shots per spectrum. TOF/TOF tandem MS fragmentation spectra were acquired for each sample, averaging 4000 laser shots per fragmentation spectrum on each of the 10 most abundant ions present in each sample (excluding trypsin autolytic peptides and other known background ions).

#### 2.5.7. Database Search

Both the resulting peptide mass and the associated fragmentation spectra were submitted to the GPS Explorer workstation equipped with the MASCOT search engine (Matrix Science) to search the database of the National Center for Biotechnology Information non-redundant (NCBInr) or Swiss-Prot-database.

Searches were performed without constraining protein molecular weight or isoelectric point, with variable carbamidomethylation of cysteine and oxidation of methionine residues. One missed cleavage was also allowed in the search parameters. Candidates with either protein score C.I.% or Ion C.I.% greater than 95 were considered significant. 

The list of proteins with levels of ratio µG/1G ≥ 2 was loaded into the Reactome pathway database. Reactome is a curated and peer-reviewed database of pathways and reactions in human biology [29]. For the analysis, we have considered only those pathways with a probability score, corrected for false discovery rate by the Benjamini–Hochberg method, <0.05.

### 2.6. Statistics

Data displayed a normal distribution (Shapiro–Wilk test) and are presented as mean ± SD. Statistical analyses for the cell markers were performed using One-Way ANOVA, followed by Tukey post hoc test in which *p* < 0.05 was defined as statistically significant.

## 3. Results

### 3.1. Live OLPs Back from Space

In the current study, we could not visualize OLPs while in space. Nonetheless, post-flight observations using time-lapse microscopy allowed us to follow in detail their behavior and recovery after harvesting and seeding them in flaskettes with glass bottom for great quality imaging. After space flight on board SpX-21, the size of the cell pellet was two times larger than that used to seed the cells onto the mesh for the passive hardware. The initial seeding cell density was 0.7 × 10^6^ OLPs per well. Upon return from space flight, we obtained 2.8 × 10^6^ OLPs per well.

**Figure 5:**
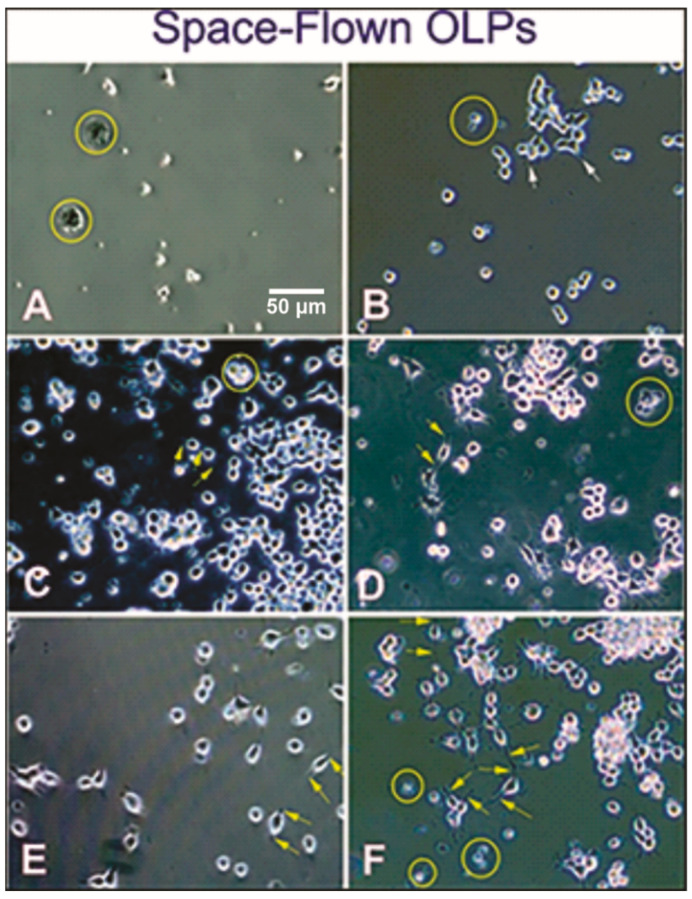
(**A**) Upon arrival, after harvesting from the mesh carriers, freshly plated OLPs that looked quiescent and devoid of cell processes and some that were presumably dying or died during the trip back to Earth (circles) 5 min after plating. They remained as such for the next 24 h. (**B**) After 48 h, more cells had adhered to the substrate and started to show tiny cell processes (white arrows). Others were round and remained adhered to the substratum. (**C**) One week after return to Earth, some OLPs tended to display a bipolar morphology, although the vast majority remained healthy, round, and without cell processes. (**D**) Ten days after being seeded, more cells displayed bipolar morphology (arrows). Most cells were alive, round, and looked healthy, and only a few were still presumably dying. (**E**) Three weeks after their return, numerous cells had a changed morphology from round to bipolar with long cell processes (arrows) and remained as single cells with virtually no cell death visible. (**F**) In the fourth week, OLPs had proliferated and continued to divide in a clonal-like manner forming clusters or OLP colonies; some appeared to be dividing. Nonetheless, a few cells were still presumably dying, suggesting that some could not re-adapt to Earth’s gravity even 4 weeks after their return, fed with fresh medium and kept in the standard conditions. Our analysis shows that human OLPs derived from hiPS survived the space flight, re-entry, and splash-down. They recovered slowly, and the vast majority were able to proliferate normally as they re-adapted to Earth’s gravity. Their proliferation rate was increased but not statistically significant with respect to GC-OLPs (Supplemental Figure S1).

Aside from time-lapse microscopy, still images were acquired to document how cells developed through time back on Earth. Two hours after plating, one T-25 flask was used for time-lapse microscopy: cells looked very ‘tired’, without cell processes, and apparently immobile. One might think that they were dead; however, they adhered to the substratum indicating that they were alive. As time elapsed, OLPs started to recover in the fresh medium. While a few cells were still floating, a small number of cells were still dying, presumably due to the stress suffered during unberth and splash-down. Nonetheless, some cells appeared to be dividing and some showed short sprout-like cell processes. Cells proliferated, most survived adhered to the substratum, and a few were still dying. Most OLPs were round or still with tiny cell processes that appeared to bear a bipolar shape. In other areas of the cell culture, most cells were round, but more cells were bipolar, and a few were multipolar. With time, cells were still dividing, and the vast majority were bipolar. OLPs remained proliferating and forming clusters of round cells. Many were bipolar, and very few presented three or four processes. In addition, some cells were still unhealthy and presumably dying (Figure 5).

### 3.2. Space-Flight Induced Incomplete Cytokinesis in OLPs

**Figure 6:**
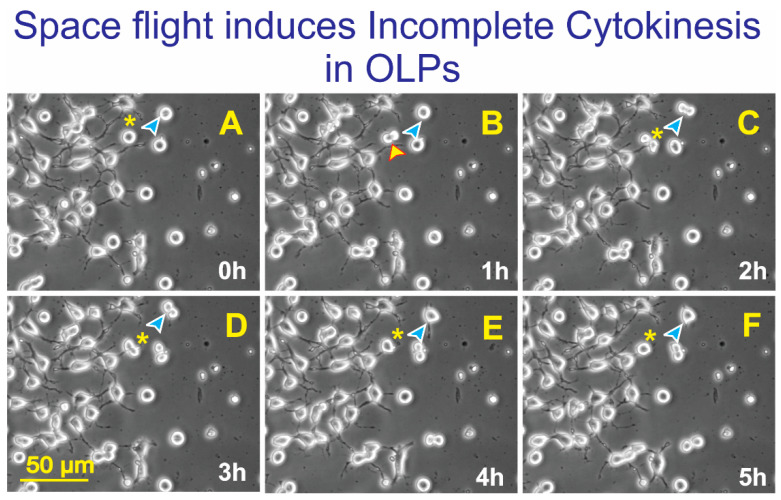
Upon return from space, OLPs were seeded onto poly-d-lysine coated flaskettes with bottom glass in the welcoming culture medium BS1. (**A**) View of cells OLP1 (asterisk) and OLP2 (blue arrowhead). (**B**) OLPs appear to be dividing (yellow arrowhead). OLP2 exhibited no change. (**C**) OLP1 showed no change, and OLP2 appears to be dividing. (**D**) OLP1 did not divide. OLP2 (blue arrowhead) did not divide either. Instead, it displayed two cell processes. (**E**) Neither of the cells divided. (**F**) After attempting to divide between hour 2 and 3 the cell remained in the same position and as a single cell. Frames were taken every 10 min.

A cell suspension of OLPs was prepared in OSM and seeded onto mesh pieces placed in the flying hardware. The devices were filled with the medium, and air bubbles were removed prior to closing them. OLPs were flown in space for 35 days and 9 h. Upon return, the cells contained in the hardware units were transferred from Kennedy Space Center to UCLA. OLPs were recovered from each well separately and seeded onto separate poly-d-lysine (PdL) coated flaskettes using OSM medium supplemented with IGF-1. We observed that the vast majority of the cells were able to proliferate normally. Nonetheless, a subpopulation showed difficulty in completing cytokinesis, even though they had successful karyokinesis. An example of such cells is shown in Figure 6.

**Figure 7:**
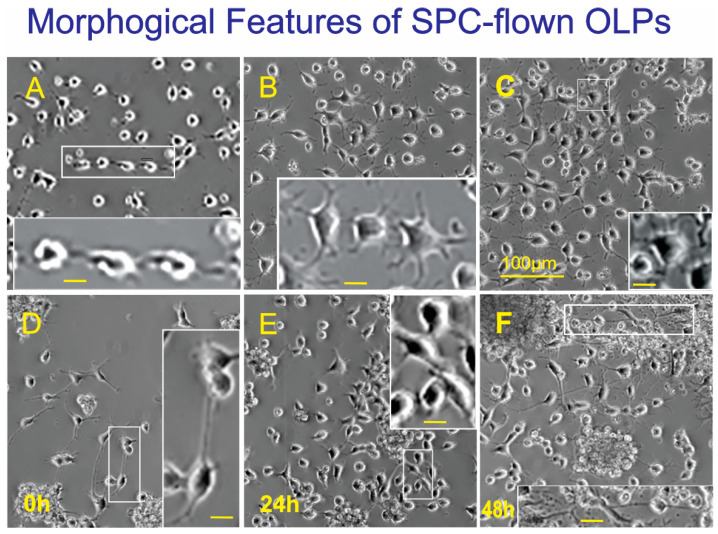
Representative views of GC (**A**–**C**) and SPC-flown OLPs (**D**–**F**). (**A**) View of the cells just after seeding. OLPs were mostly round or bipolar (inset). (**B**) Twenty-four hours after, while many cells were bipolar, there were also cells bearing 3 to 5 cell processes (inset). (**C**) Most OLPs displayed multiple thin cell processes 48 h after the time-lapse started. (**D**) SPC-flown OLPs after being harvested from the mesh carriers, where most of them were bipolar or tripolar bearing hair-thin cell processes. Bipolar cells presented very long cell processes, reminiscent of radial glia. (**E**) After 24 h, the vast majority of the cells were bipolar or tripolar. They appeared to form chains by interconnecting with their cell processes (inset). (**F**) Some of the cells had formed clusters composed of round cells without processes. Other OLPs were in rosary-like formations, and the bipolar cells extended their processes connecting other cells or clusters (inset). Inset scale bar = 10 µm.

We compared the SPC-OLPs and GC OLPs-cultures during the first week. Twenty-four hours after having been plated onto flaskettes (at the beginning of the experiment or time 0 h), we found that GC-OLPs started becoming bipolar. However, as soon as 24 h later, some of these cells had become multipolar, and they moved forward in the lineage to display numerous hair-like processes that interconnected with other cells’ processes. SPC-OLPs cells were bipolar when alone and bore longer cell processes than GC cells, reminiscent of radial glia. Some cells formed clusters, where the majority did not show cell processes aside from those in direct contact with the substratum. After 48 h, they appeared to prefer growing in clusters adhered to each other rather than the substratum. Nonetheless, those growing as single cells were round and devoid of processes. Bipolar OLPs continued to have radial glia-like long processes (Figure 7). 

**Figure 8:**
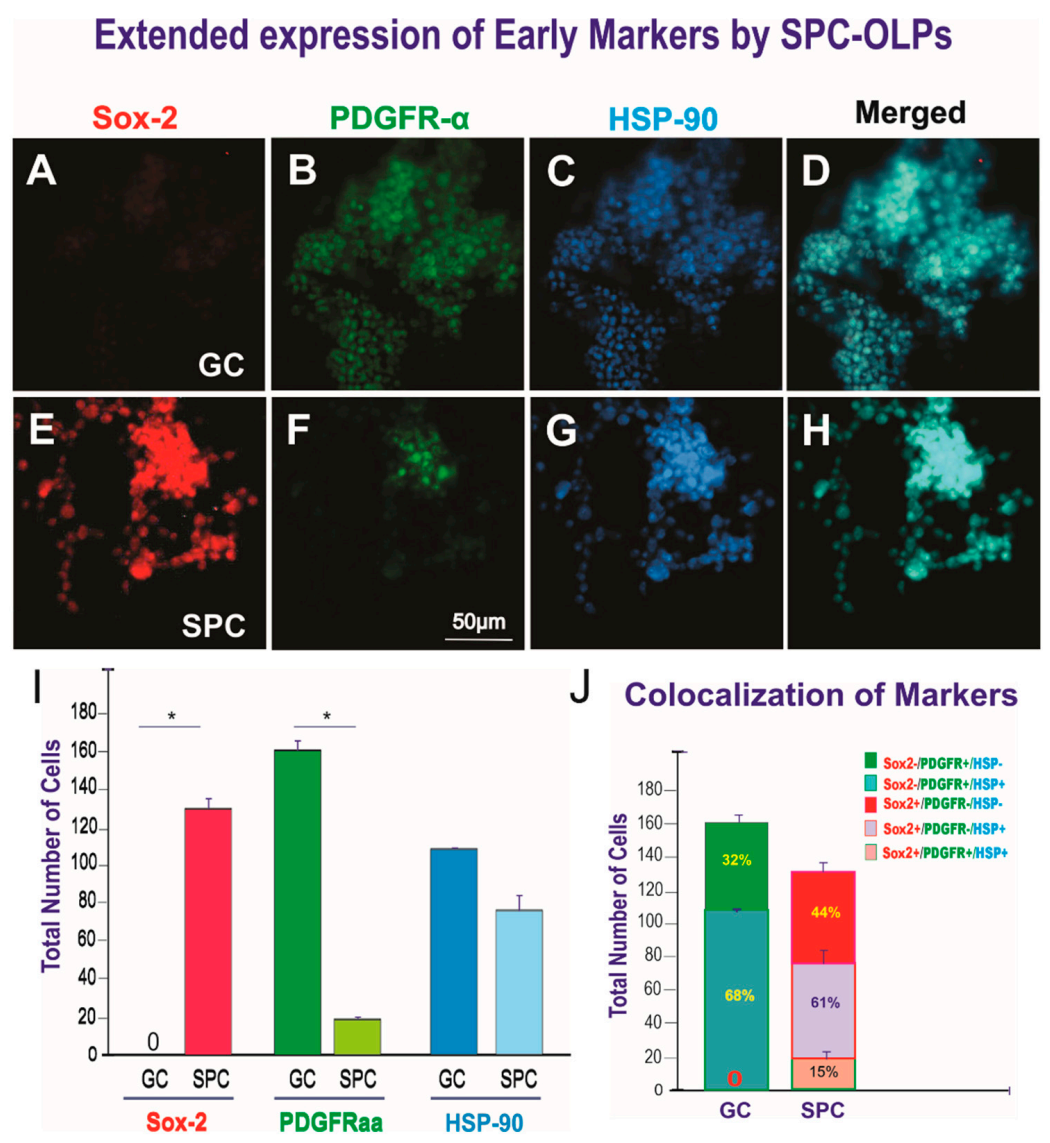
Views of the expression of Sox-2, PDGFR-α, and HSP-90 by OLPs. (**A**–**C**) Ground control (GC). (**D**–**F**) SPC-flown OLPs. (**A**) OLPs did not express Sox-2. (**B**) The vast majority expressed PDGFR-α. (**C**) They also expressed HSP90β, indicating that they were still recovering from having been unattended in the flying hardware. (**D**) View of the three markers merged. (**E**) Sox-2 was intensely expressed by most space-flown OLPs. (**F**) Only a subset of SPC-OLPs co-expressed PDGFR-α. (**G**) The vast majority of these cells expressed HSP-90β. (**H**) View of the three markers merged. (**I**) The bar graph shows the comparison of the expression of Sox-2, PDGFR-α, and HSP-90β by OLPs in GC or SPC-flown OLPs (SPC). GC OLPs did not express Sox-2 expressed in most OLPs. In contrast, many SPC-OLPs were positive for this early marker of OLPs. In the case of PDGFR-α, the vast majority of GC-OLPs were positive, while only a small number started expressing it. Interestingly, the differences in HSP-90β expression were not statistically significant, and there were more positive OLPs in the GC group when compared to SPC-flown OLPs, suggesting that having been left unattended for 45 days in the same culture medium triggered HSP-90β expression regardless of gravity (Earth vs. space gravity). Data were plotted as the mean of four fields. Statistical significance was assessed by one-way ANOVA, in which *p* < 0.05 was defined as statistically significant (*) *p* < 0.01. (**J**) This bar graph shows the expression of markers alone or their colocalization. In the GC group, 68% of cells co-expressed the stress protein HSP90 and PDGFR-α that is a marker expressed by more mature progenitors than those that express Sox2, which was negative in this group. Not all cells expressed HSP-90, as 32% expressed only PDGFR-α. For SPC-OLPs, HSP90β colocalized with the immature marker Sox2 reaching 68%, and only a small percentage, 15%, co-expressed the three markers. Only 44% of these progenitors expressed Ferritin alone. The data are shown both in cell numbers and percentages. Statistical significance was assessed by one-way ANOVA, in which *p* < 0.05 was defined as statistically significant (*) *p* < 0.01.

Next, we examined the expression of specific OLP markers to ascertain how space microgravity would affect their ontogeny. OLPs were cultured during one week prior to performing triple immunofluorescence. Our data showed that control OLPs that grew in the flying hardware but remained on Earth did not express the early marker Sox-2 while already expressing PDGFR-α, indicating that despite remaining unattended, they survived and expressed this typical OLP marker as expected. To determine if staying 45 days unattended would stress them, we also labeled them for HSP-90β and found a mild expression of this protein. In contrast, all SPC-flown OLPs expressed Sox-2, while only a few of them expressed PDGFR-α, suggesting that they were slightly more immature than their GC counterparts. Moreover, these cells co-expressed HSP-90β, indicating that they survived and expressed early markers of OLPs despite the stress inflicted by space flight. The cell counts of Sox-2 and PDGFR-α showed a delayed expression of markers by SPC-flown OLPs. The former is known to appear at an earlier developmental stage in the lineage, and the latter is known to be expressed by less immature OLPs. In addition, both GC and SPC-OLPs expressed HSP-90β (Figure 8). Results in percentages are shown in Supplemental Figure S2.

**Figure 9:**
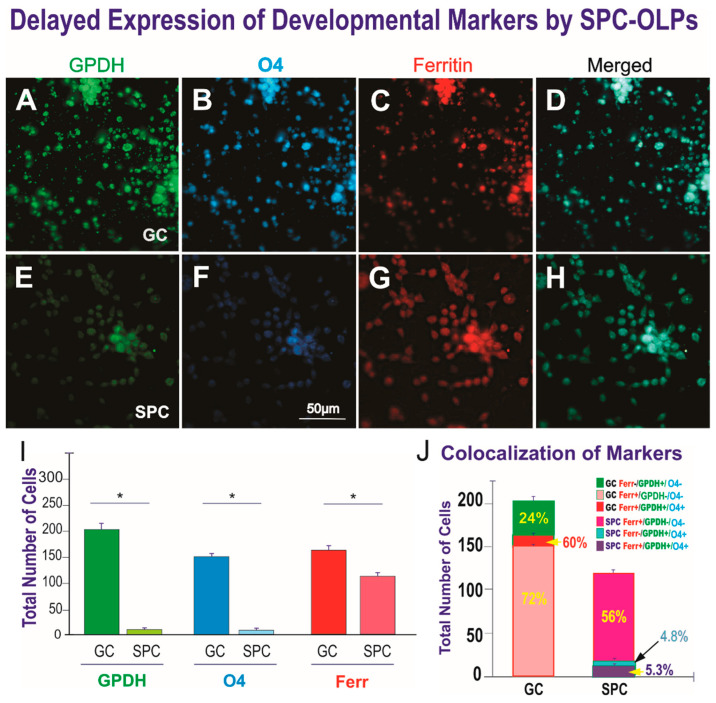
Views of the expression of GPDH, O4, and ferritin by OLPs. (**A**–**C)** Ground control (GC). (**D**–**F)** SPC-flown OLPs. (**A**) GPDH was intensely expressed by most GC-OLPs. (**B**) These cells were co-labeled by the antibody O4. (**C**) They also expressed ferritin that labels these cells during development. (**D**) Merged view of the three markers expressed. (**E**) Only a few of the SPC-OLPs in these cultures expressed GPDH. (**F**) The number of cells expressing O4 was also reduced. (**G**) Most SPC-NSCs were labelled for ferritin. (**H**) Merged view to visualize the colocalization of the three markers. (**I**) This bar graph shows the expression of the OLP markers GPDH, O4, and ferritin expressed by ground control (GC) and SPC-flown OLPs (SPC). The first two markers were expressed in most OLPs. In contrast, their expression by SPC-OLPs was limited to very few cells. Ferritin was expressed by OLPs under the same conditions, and although there were more Ferr-positive cells in the GC group, at least two thirds of the SPC-OLP group expressed this protein. Results are from four separate fields of view. A total of 1800 cells per condition were counted. Data are reported as mean of 4 fields of view ± SD (* *p* < 0.01). Statistical significance was assessed by one-way ANOVA, in which *p* < 0.05 was defined as statistically significant * *p* < 0.01. (**J**) This bar graph shows the percentage of the markers expressed alone or co-expressed by OLPs. In the GC group, 72% co-expressed the three markers, while only 60% co-expressed both OL markers but not the stress protein. Only 24% of these cells expressed GPDH alone. In the case of SPC-OLPs, only 5.3% co-expressed the tree markers, indicating the immature nature of these cells. GPDH and HSP90 were found in 4.8% of SPC-OLPs, and 56% of space-flown cells expressed only ferritin. The data are shown both in cell numbers and percentages. Statistical significance was assessed by one-way ANOVA, in which *p* < 0.05 was defined as statistically significant (*) *p* < 0.01.

In order to ascertain if OLPs remained functional and progressed in their developmental lineage, we examined the expression of the GPDHR-α two weeks after cells were plated. With time, a majority of GC-OLPs intensely expressed this marker specific for the next developmental stage in their somata. It was co-expressed with O4, a marker that defines these cells as pre-myelinating OLs. This is important because it labels sulfatides required for the transition of OLPs to young OLs to start synthesizing myelin components. In the case of SPC-OLPs, only a few cells had begun to express this marker, suggesting a delay in the development of SPC-OLPs. Nonetheless, both groups, GC and SPC-OLPs, had remained functional and were moving forward in their lineage as expected. Moreover, since OLs are also involved in iron metabolism, in the CNS, we examined ferritin, the iron storage protein, and it was expressed by both GC and SPC-OLPs. (Figure 9). Results in percentages are shown in Supplemental Figure S3.

**Figure 10:**
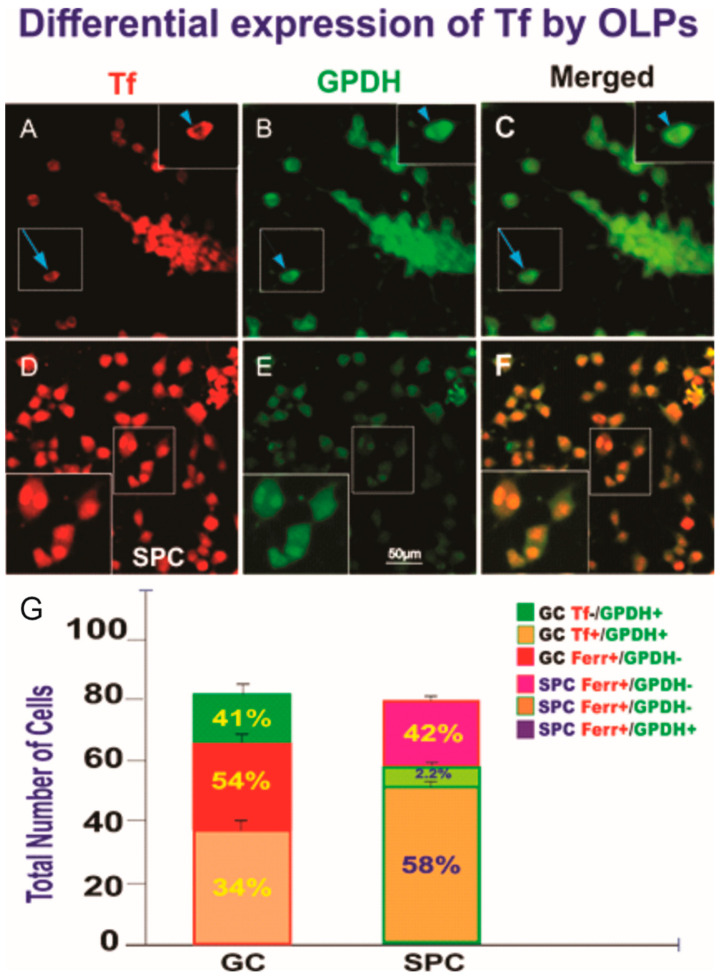
Views of GC-OLPs (**A**–**C**) or SPC-OLPs (**D**–**F**) two weeks after being seeded. (**A**) Most GC-OLPs expressed Tf in their cytoplasm (inset and arrow). (**B**) They also co-expressed GPDH. (**C**) Merged view of both markers that colocalized. (**D**) Tf was expressed by all cells in their nuclei rather than the cytoplasm. (**E**) Most of these cells were weakly labeled for GPDH, and only those that had undergone karyokinesis or in the process of cytokinesis displayed nuclear GPDH expression. (**F**) Merged view of both markers. (**G**) This bar graph shows the percentage of the markers expressed alone or co-expressed by OLPs. In the GC group, 34% co-expressed both OL markers. Only 51% expressed transferrin alone, and 41% expressed GPDH only. In the case of SPC-OLPs, only 58% co-expressed Tf in the nucleus and cytoplasmic GPDH, although the GPDH expression was faint and it colocalized in the nucleus only in cells that had undergone karyokinesis and those that appeared to be undergoing cytokinesis. Only 2.2% of these OLPs expressed GPDH alone, indicative of their immature stage with respect to GC cells. The data are shown both in cell numbers and percentages. Statistical significance was assessed by one-way ANOVA, in which *p* < 0.05 was defined as statistically significant (*p* < 0.01. An orthogonal image of the localization of Tf is shown in Supplemental Figure S4.

Based on the results described above, where most SPC-OLPs did not express GPDH or O4, we examined the expression of transferrin (Tf), which we had previously described as an early marker for OLPs, and we found that Tf was expressed by both GC and SPC-OLPs. Nonetheless, we found a very important difference. GC-OLPs expressed this protein in their cytoplasm, while in SPC-OLPs, its expression was found in the nucleus (Figure 10).

The examination of the secretome of SPC-flown OLPs using mass spectrometry-based quantitative proteomics revealed that Tf was the second most abundant protein contained in the secretome of these cells (Table 3).

**Figure 11:**
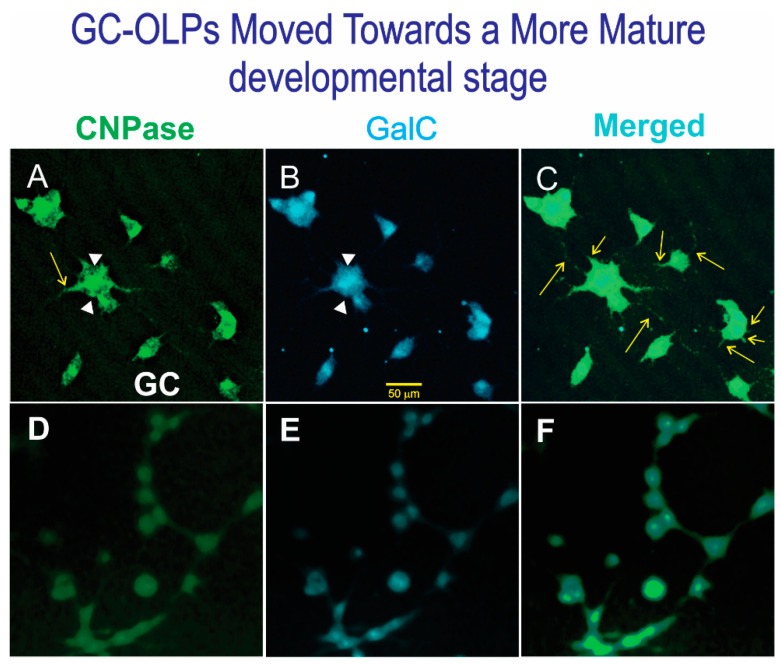
Views of GC-OLPs (**A**–**C**) or SPC-OLPs. (**D**–**F**) Cells were fed for two weeks. (**A**) Most GC-OLPs expressed CNPase, and the morphology of the cells indicates that GC cells had moved towards the next developmental stage as they had developed multiple cell processes in two weeks after being cultured in OLDEM with IGF-1 and T3. (**B**) They also co-expressed GalC (the arrowheads point to the thin membrane adhered to the substrate). (**C**) Merged view of both markers that colocalized (the arrows point to cell processes). (**D**–**F**) SPC-OLPs did not express either CNPase or GalC. The cells were either round or bipolar after 7 weeks in culture (5 weeks with OSM supplemented with Tf + IGF-1 and 2 weeks in OLDEM Tf + IGF-1)**.** (Views of control serum to assess background noise for the three fluorophores are shown in Supplemental Figure S5).

We next examined if space-flown OLPs would respond to a culture medium known to induce a more mature phenotype. To do so, we fed the cells with welcoming medium supplemented with Tf and kept them in this medium for five weeks. SPC-flown OLPs did not undergo significant changes; they remained bipolar or round. After this trial, we replaced the medium with OLDEM medium that we have previously described to support the maturation of OLs. When we did not see any changes after 10 days, we decided to supplement OLDEM with IGF-1 and T3. We kept monitoring the cells for morphological changes and fed them with this medium for five more weeks. At the end of the experiment, we examined the cells for expression of CNPase and GalC expression, despite their exposure to media known to induce their mature, myelinating phenotype (Figure 11). A diagram showing the developmental stages supported by each culture medium has been published previously [9].

## 4. Discussion

The fast propagation of electric impulses in the CNS is possible thanks to the myelin sheath. Myelin makes saltatory conduction in nerve fibers possible through the nodes of Ranvier. It is vital to have a source of OLPs that mature and perform the specialized function of producing and maintaining myelin. CNS myelin disorders are a public health challenge due to the lack of adequate therapies. Two of the most notable of these conditions are brain white matter injury (WMI) in premature infants and multiple sclerosis (MS) in adults [1]. Myelin does not regenerate spontaneously; therefore, to develop new therapeutic strategies to be used on Earth and countermeasures for astronauts in long-term space missions, it is critical to understand the molecular mechanisms underlying both OL development and myelination, as well as their interaction with neurons while in microgravity.

One of the characteristics of OLs’ development is that they undergo specific morphological changes along with concomitant expression of specific cell markers as they move forward on the lineage. These stages are well known under Earth’s gravity. Nonetheless, how space flight influences their development as they move towards maturation and myelination has not been studied. Here, we show that OLPs back from space undergo transient changes as they adapt to Earth’s gravity, including round cell bodies without cell processes. Yet, once adapted to the environment, OLPs recapitulate morphological cues such as bipolar morphology typical of developing OLPs and normal proliferation. Nonetheless, a subpopulation of OLPs showed difficulties in dividing, resulting in incomplete cytokinesis most likely due to the effects of mechanical forces on cytoskeleton remodeling. We show an extended expression of the early OLP marker Sox2 with a concomitant delayed expression of PDGFR-α, GPDH, and the glycosphingolipid GalC, essential for the synthesis of sulfatides, a major building block for myelin. Moreover, the main permanent change we show is that when these cells were fed with culture media designed to support the synthesis of enzymes such as CNPase to initiate the machinery necessary for the synthesis of glycolipids and proteolipids, they failed to move forward on the lineage. All these signs are indicative of the immature nature of SPC-OLPs after exposure to microgravity. Using the stress protein HSP-90, we also show that the space flight was not detrimental to the cells and that more GC cells expressed this protein with respect to SPC-OLPs. Being unattended for 45 days increased the expression of HSP-90 in GC cells, and that in microgravity cells appeared more resistant to stress.

In addition, we discovered the nuclear expression of Tf in most, if not all, SPC-OLPs, which may have implications for iron metabolism, OLPs’ lineage progression, and myelination. Moreover, we demonstrated that Tf and IGF-1, despite their known robust trophic support and synergistic effects on myelination in vivo and in culture (Espinosa et al., 2013, 2016), did not influence the immature status of SPC-OLPs. Furthermore, we have previously reported that when Tf is removed from the culture medium, OLs move forward to a myelinating stage expressing myelin markers [30]. Nonetheless, when SPC-OLPs were exposed to OLDEM containing IGF-1 and T3 for two weeks, they did not move to the next developmental stage, as if they remembered that they had been subject to microgravity. In addition, although the global proteomics analysis of the secretome of SPC-OLPs is still ongoing, we hereby can report that Tf is the second most abundant protein in it. Whether the high levels of Tf in the secretome or its localization to the nucleus of these cells is what prevents OLPs from maturing remains to be ascertained.

Time-lapse microscopy also allowed us to ascertain that some of these SPC-OLPs had difficulty adapting to Earth’s gravity; this was visualized when cells appeared to pull the dividing cell to assist cell division. In some cases, the cell could not complete cytokinesis even though karyokinesis was successful, giving rise to a subpopulation of OLPs with two nuclei resulting in tetraploid cells. This process on Earth is considered abnormal in most mammalian cell systems and organs and, in some cases, may be deleterious and could be a feature of cancer cell precursors [31]. There is evidence that cell shape and architecture depend mainly on the cytoskeleton [32]. It is also known that the cytoskeleton components and their organization are essential during the cell cycle, particularly during mitosis for the completion of cell cleavage [33]. In the present study, taking together increased proliferation and abnormal cytokinesis, we could infer that increased OLP proliferation and incomplete division after space flight may be explained by cytoskeleton changes that may have accelerated karyokinesis and cytokinesis while in microgravity. Still, under the forces of gravity, OLPs encountered resistance by their stronger adhesion to the substrate and failed to complete cytokinesis in some cases.

To our knowledge, there are no studies on the mechanical properties of OLPs in microgravity. Nonetheless, a study focusing on the effects of sim-µG using endothelial cells (ECs) showed a significant reduction in their membrane viscosity and elasticity after 24 h exposure to sim-µG. Both properties are essential to maintain a functional equilibrium of the vasculature, whose failure leads to atherosclerosis and other pathologies. Sim-µG reduced ECs’ rigidity leading to rounding of the cells. Moreover, cytoskeletal components were reduced, and cells’ immunocytochemistry revealed disorganization of both actin filaments and microtubules that altered ECs’ morphology, cell behavior, and function, including proliferation, differentiation, signaling, gene expression, surface adhesion molecules, and extracellular matrix protein expression [34,35]. This is important because microgravity leads to the association of orthostatic intolerance with syncope, increase in resting heart rate, and decrease in physical capability syndrome known as cardiovascular deconditioning [36,37]. Our previous and the current study show that OLPs are gravity sensing cells and that weightlessness also impacts OLPs’ proliferation, lipid metabolism, and, as shown here, lineage progression (Espinosa et al., 2013; Espinosa et al., 2016). In the case of OLPs and mature OLs, membrane fluidity, lineage progression, and cytoskeleton organization are paramount as they produce the myelin sheath, whose formation depends on transport of proteins and mRNA to their numerous cell processes as the first step of myelination. Therefore, future studies on the viscoelastic and mechano-transduction properties of OLPs and OLs while in microgravity and after exposure to it are necessary to devise countermeasures to preserve astronauts’ health. 

Incomplete cytokinesis was not the focus of the present study, but given the immense diversity of cell cycle regulation, it raises many questions, and it will be the subject of future investigation. It is essential to understand to which extent this is temporary cell cycle remodeling [38] while adapting from microgravity exposure to Earth’s gravity or a sign of potential malignancy due to radiation exposure. It is also a matter of extensive exploration. 

We have previously described that the iron carrier glycoprotein Tf is an early marker for OLPs [39], where all OLPs start by expressing Tf, and as they move forward on the lineage, they form three subpopulations in adulthood where one subpopulation expresses only Tf, another group expresses both myelin basic protein (MBP) and Tf, and a third group expresses MBP only [40,41]. We also studied by run-off transcription the site of Tf action for the expression of the MBP-gene, and we found that MBP mRNA was significantly increased at the nuclear level (Espinosa et al., 2002). Based on these data, we added Texas red covalently attached to exogenous Tf to visualize its location in OLs, and it localized to the nucleus of several of these cells. Despite all the data supporting a direct interaction of Tf on the MBP gene at the nuclear level, we could only rarely visualize it directly in the nucleus by confocal microscopy. Here, while studying the ontogeny of OLPs after exposure to space flight, we used anti-Tf antibody as one of their typical early markers, and we found that most, if not all, SPC-OLPs exhibited Tf expression in the nucleus.

This phenomenon may have developmental implications. Although all OLPs expressed Tf during development, some lost this expression as they progressed in their lineage and began to express MBP as they matured, myelinating OLs. We currently do not have more SPC-OLPs, and therefore, new funding will be crucial to study this phenomenon. We have shown that in Earth’s gravity, a single intraparenchymal injection of Tf makes OLPs from myelin-deficient rats move forward by increased transcription and translation of MBP leading to myelination [42]. There is also evidence that insulin-like growth factor (IGF-1) is a potent regulator of OL development by increasing the numbers of OLPs via proliferation and through maturation in cell culture [43] it also enhances the myelinogenic properties of OLs [44]. We have also shown that the combination of Tf and IGF-1 supports myelination in a mouse model of periventricular leukomalacia [45] and the demyelinated adult brain [13].

Some studies have shown that learning is associated with changes in white matter and oligodendrogenesis. It has also been shown that inhibition of OLs’ differentiation impairs learning in a matter of hours [46]. Moreover, spatial learning promotes neurogenesis, oligodendrogenesis, and new myelin formation in adult mice. In addition, reduced or impaired oligodendrogenesis compromises spatial memory consolidation by blocking spatial learning hippocampal ripple-cortical spindle coupling [47]. Thus, changes in myelination and myelin structure significantly affect human behavior. Nonetheless, a direct link between myelin plasticity and behavior has not yet been established. Therefore, understanding how to make OLPs move forward to functional myelinating OLs is of utmost importance. There are several therapies for MS [48], but none of them reverses or stops the neurodegenerative damage, protects naked axons, or triggers sustained re-myelination [49]. In MS, there are clinical deficits such as gait, semantics, phonetics, and auditory, as well as irreversible cognitive deficits, such as episodic memory and processing speed, which are critical components of working memory [50]. Thus, cellular, molecular, and behavioral studies are still needed to address MS in a holistic approach. The present work shows that microgravity offers a novel platform to start unraveling the cellular and molecular mechanisms that entail the well-known plasticity of the CNS in health and disease. Our findings provide good evidence of how gravitational changes may lead to the identification of molecules, mechanisms, and their regulation, an unimaginable situation just a few decades ago. The new knowledge will be a valuable tool to address MS and myelin loss due to injury and to understand changes that may be generated by microgravity and radiation in the space environment.

As expected in all pioneer work, there are some inherent limitations in our study. Although we primarily focused on the effects of microgravity/space flight on OLPs, other factors may contribute to the changes we observed. Therefore, future studies will have to include the effects of radiation on OLP proliferation and growth in our SPCs compared to ground controls. For example, it is worth mentioning that experimental studies have shown that high atomic number energy (HZE) nuclei produce both qualitative and quantitative differences in biological effects compared to terrestrial radiation [51], leading to uncertainty in predicting exposure health outcomes in humans. Recent research has also shown that low dose-rate exposures to neutron irradiation produce serious neurocognitive disturbances associated with impaired neurotransmission. Chronic (6 month) low-dose (18 cGy) and dose rate (1 mGy/) exposures of mice to a mixed field of neutrons and photons resulted in diminished hippocampal neuronal excitability and disrupted long-term potentiation with severe impairments in learning, memory, and concomitant distress behaviors [52]. In the case of OLPs in an in vitro model of OLPs exposed to protons/HZE radiation, alterations in DNA repair protein Apurinic Endonuclease-1(APE1) expression in the nuclei and mitochondria have been described [53]. Moreover, ionizing radiation (IR) that is used to treat central nervous system (CNS) tumors has been reported to cause impaired OLPs’ differentiation and maturation as well as a decreased number of mature OLs, leading to white matter loss and impaired cognition [54]. Another report has shown that fractionated radiation exposure of rat spinal cord leads to latent neuro-inflammation in the brain, accompanied by cognitive deficits, with alterations in APE1and OPC differentiation [55]. Considering that myelin is essential for neuronal function, we have started to address the effects of low dose radiation on OLPs. In preliminary studies we exposed HuOLPs to 20cGy gamma radiation to test their viability and found that control cells appeared bright, healthy, and well adhered to the substrate. In contrast, after gamma radiation, about 25% of OLPs were lost, but sufficient cells had recovered and displayed thin short processes. 

## 5. Conclusions

This is the first study examining the effects of microgravity on OLPs flown into space. We demonstrated that SPC-OLPs remain immature, as they continued to express immature markers. Importantly, Tf displayed ectopic and sustained expression in the nuclei of SPC-OLPs, suggesting that it could interfere with normal maturation potentially via inhibition of MBP. Based on these data, we postulate that Tf translocation to the nucleus might be post-translationally regulated in response to microgravity. Nonetheless, it is not known if it remained in the nucleus as the MBP gene regulator or if it is controlling other oligodendrocyte/myelin related genes and/or iron related genes, such as ferritin and transferrin receptor. These are all fascinating topics for further investigation.

For future directions, experiments that come to mind include: (i) to ascertain the metabolic changes produced on OLPs derived from human induced pluripotent stem cells (hiPS) *acutely* exposed to sim-µG and fractionated 18–30 cGy gamma/X-rays in acute vs. chronic exposure; (ii) to compare the global methylome profiles from hiPS-derived OLPs *chronically* and *simultaneously* exposed to sim-µG and X-ray + C-ions. These studies have been submitted for consideration for funding. In conclusion, the present data allow us to conclude that microgravity can serve as a novel platform to study why in some myelin disorders OLPs are present but do not mature. It is possible that manipulations designed to rectify ectopic expression of Tf will allow proper maturation of OLPs, making it relevant to myelin diseases.

## Figures and Tables

**Table 1:** Preparation of OSM basal.

Reagents	Measurements
Insulin	5 mg
Transferrin	50 mg
Putrescine	16.1 mg
Sodium Bicarbonate	2.2 g
* Sodium Selenite	4 µL
D( + ) galactose	4.6 g
Kanamycin	500 mL

* Prepare a “stock solution” 0.8 mg/mL in PBS. Add 4 µL of the stock solution for 500 mL medium. Adjust the pH to 7.4 (after filtering).

**Table 2:** Timetable of the activities performed upon arrival of the cells to the International Space Station.

Activity	Time (GMT)	Time (PDT)
SpX21 Launch	GMT 341 at 16:17 GMT	6 December 2020 @ 08:17
Installation of Ecs into	GMT 343:14:44 (2020)	8 December 2020 @ 06:44
STaARS-1 EF		
First media exchange	GMT 345:16:32 (2020)	10 December 2020 @ 08:32
2nd media exchange	GMT 008:15:09 (2021)	8 January 2021 @ 07:09
Removal of 4 Ecs from EF-1	GMT 008:15:09 (2021)	8 January 2021 @ 07:09
SpX21 Undock	GMT 012 at 14:05 GMT	12 January 2021 @ 06:05
SpX21 Splashdown	GMT 014 at 1:26 GMT	13 January 2021 @ 16:26
Samples’ arrival at UCLA	GMT 015: 15:30 (2021)	15 January 2021 @ 4:30 am

**Table 3:** Accession information showing the abundance of Tf that was significantly up-regulated in SPC-OLPs.

Relativity	Accession No.	Gene	Top Ranked Protein Name [Species]
5.9	TRFE_HUMAN	TF	Serotransferrin OS = Homo sapiens OX = 9606 GN = TF PE = 1 SV = 3
39.27	TRFE_HUMAN	TF	Serotransferrin OS = Homo sapiens OX = 9606 GN = TF PE = 1 SV = 3
8.47	TRFE_HUMAN	TF	Serotransferrin OS = Homo sapiens OX = 9606 GN = TF PE = 1 SV = 3
8.72	TRFE_HUMAN	TF	Serotransferrin OS = Homo sapiens OX = 9606 GN = TF PE = 1 SV = 3
15.59	MEAN		

## Data Availability

All data obtained from this study has been included in the manuscript.

## Supplementary Materials

The following supporting information can be downloaded at: https://www.mdpi.com/article/10.3390/life12060797/s1, Figure S1: SPC-OLPs Proliferate more than GC OLPs. Figure S2: Percent of Positive cells between GC and SPC-OLPs for the early marker Sox2 and PDGFR-α and HSP90. Figure S3: Percent of Positive cells between GC and SPC-OLPs for GPDH, O4 and Ferr. Figure S4: Transferrin Localizes to the Nucleusof space-flown OLPs. Figure S5: Views of the control serum for detection of fluorescent background from the fluorophores used. Table S1: Tally of activities performed onboard the International Space Station.
