# Correction: Tran et al. Delayed Maturation of Oligodendrocyte Progenitors by Microgravity: Implications for Multiple Sclerosis and Space Flight. *Life* 2022, *12*, 797

**DOI:** 10.3390/life13102051

**Published:** 2023-10-13

**Authors:** Victoria Tran, Nicholas Carpo, Sophia Shaka, Joile Zamudio, Sungshin Choi, Carlos Cepeda, Araceli Espinosa-Jeffrey

**Affiliations:** 1 Department of Psychiatry, Semel Institute for Neuroscience and Human Behavior, The University of California Los Angeles, Los Angeles, CA 90095, USA; victoriakimtran@gmail.com (V.T.); npgcarpo@gmail.com (N.C.); shaka@g.ucla.edu (S.S.); joilezmd@gmail.com (J.Z.); ccepeda@ucla.edu (C.C.); 2 KBR, NASA Ames Research Center, Moffett Field, CA 94035, USA; sungshin.y.choi@nasa.gov

## Error in Figure

The authors wish to make the following corrections to Figure 10 of this paper [1]:

In the original publication, there is a mistake in the bar graph of Figure 10 as published.

At the moment of uploading the manuscript, we chose the wrong legend version. It should say Tf for transferrin (instead of Fer for ferritin, as published). The figure as is makes no sense because this section is about the two specific markers Tf and GPDH. Swapping the figure will correct the information without altering the data, contents or caption. The text and caption for the entire figure discuss transferrin (Tf) and the correct graph is pasted below.

**Figure 10:**
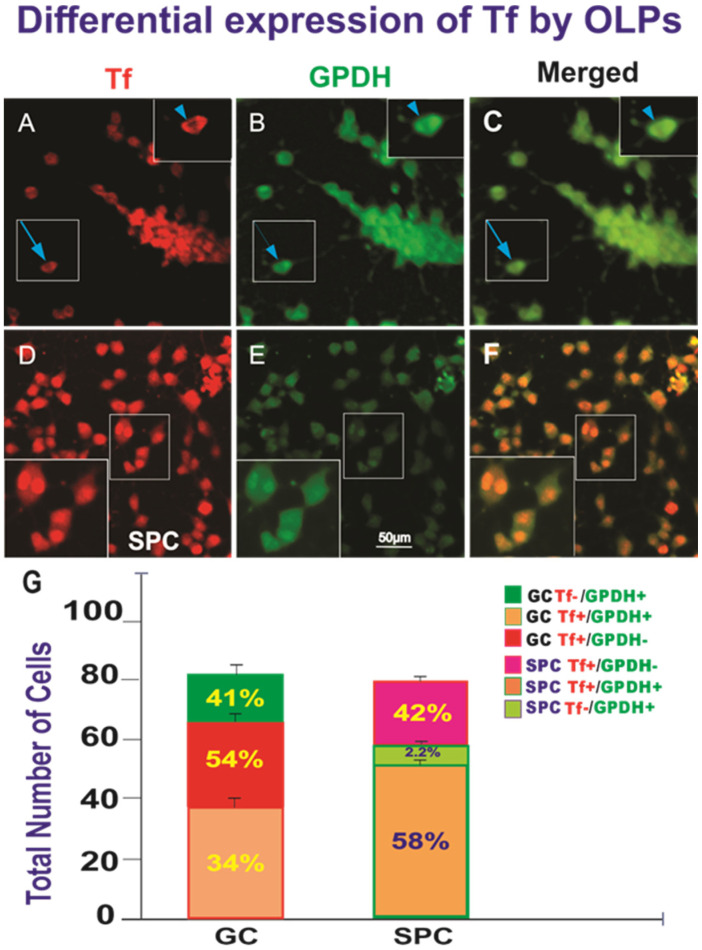
Views of GC-OLPs (**A**–**C**) or SPC-OLPs (**D**–**F**) two weeks after being seeded. (**A**) Most GC-OLPs expressed Tf in their cytoplasm (inset and arrow). (**B**) They also co-expressed GPDH. (**C**) Merged view of both markers that colocalized. (**D**) Tf was expressed by all cells in their nuclei rather than the cytoplasm. (**E**) Most of these cells were weakly labeled for GPDH, and only those that had undergone karyokinesis or in the process of cytokinesis displayed nuclear GPDH expression. (**F**) Merged view of both markers. (**G**) This bar graph shows the percentage of the markers expressed alone or co-expressed by OLPs. In the GC group, 34% co-expressed both OL markers. Only 51% expressed transferrin alone, and 41% expressed GPDH only. In the case of SPC-OLPs, only 58% co-expressed Tf in the nucleus and cytoplasmic GPDH, although the GPDH expression was faint and it colocalized in the nucleus only in cells that had undergone karyokinesis and those that appeared to be undergoing cytokinesis. Only 2.2% of these OLPs expressed GPDH alone, indicative of their immature stage with respect to GC cells. The data are shown both in cell numbers and percentages. Statistical significance was assessed by one-way ANOVA, in which *p* < 0.05 was defined as statistically significant (*p* < 0.01). An orthogonal image of the localization of Tf is shown in Supplemental Figure S4.

The authors state that the scientific conclusions are unaffected. This correction was approved by the Academic Editor. The original publication has also been updated.
