# Supplementary material for: Delayed Maturation of Oligodendrocyte Progenitors by Microgravity: Implications for Multiple Sclerosis and Space Flight
Source: Life (Basel). 2022 May 27;12(6):797. doi: 10.3390/life12060797 (PMC9224676; doi:10.3390/life12060797)
Supplement: Supplementary file 1 [file life-12-00797-s001.zip › life-1687806-supplementary.pdf]

Supplemental Figures.

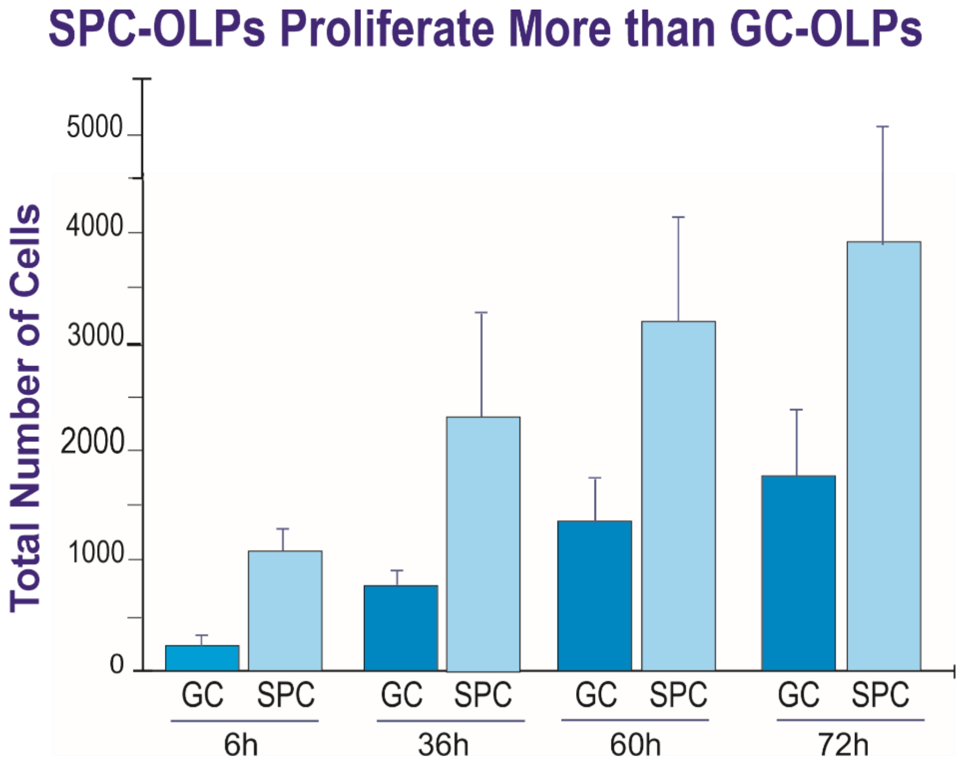

Supplemental Figure S1. After space flight, OLPs proliferated more than GC cells although the differences were not significant. Data were plotted as the mean of four fields. Statistical significance was assessed by one-way ANOVA, in which $p < 0.05$ was defined as statistically significant.

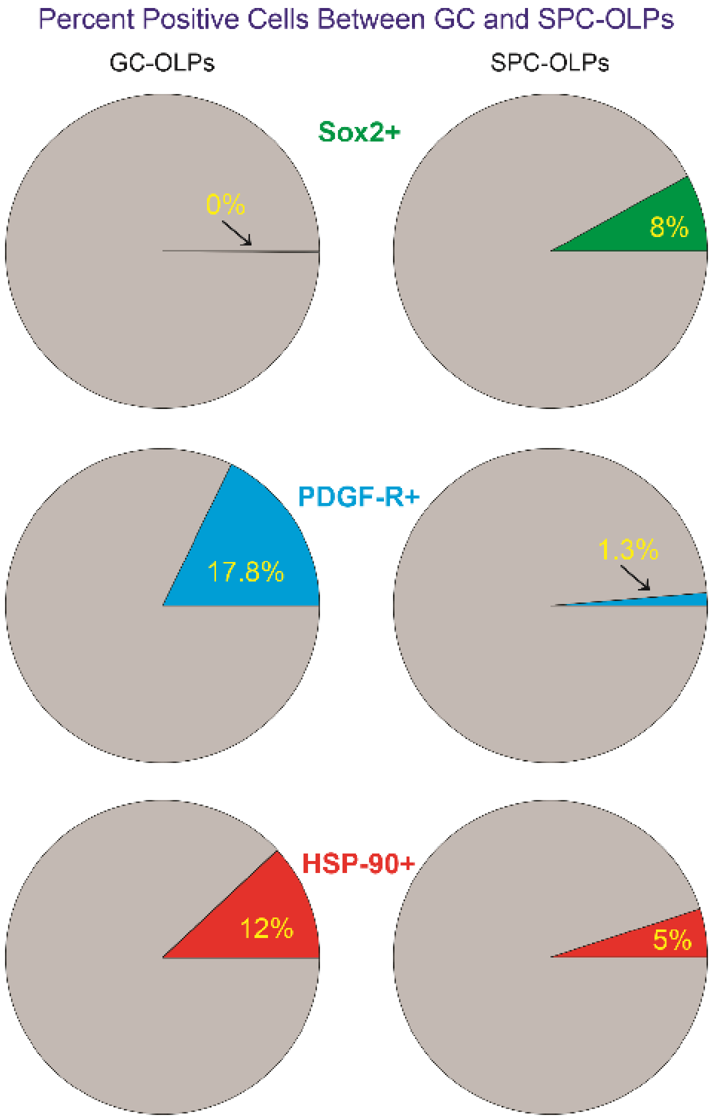

Supplemental Figure S2. View of the expression of OLP markers by GC-OLPs (left panels). The early marker Sox2 in GC-OLPs was null, while 8% of SPC-OLP expressed it. A marker expressed by OLPs that indicates that they have moved forward in their development is PDGFR alpha, almost 18% of GC-OLPs expressed it. In contrast, only 1.3% of OLPs flown onto space was positive for this marker indicating their degree of immaturity. The expression of the stress marker HSP-90β was relatively low in both groups. It appears that having been unattended on Earth for 45 days affected 12% of GC-OLPs, whereas only 5% of SPC-OLPs was positive for this marker suggesting that either they recovered faster than their GC counterparts or that microgravity might have protected them both from having been unattended and from the stress produced on them by re-entry and of splashdown.

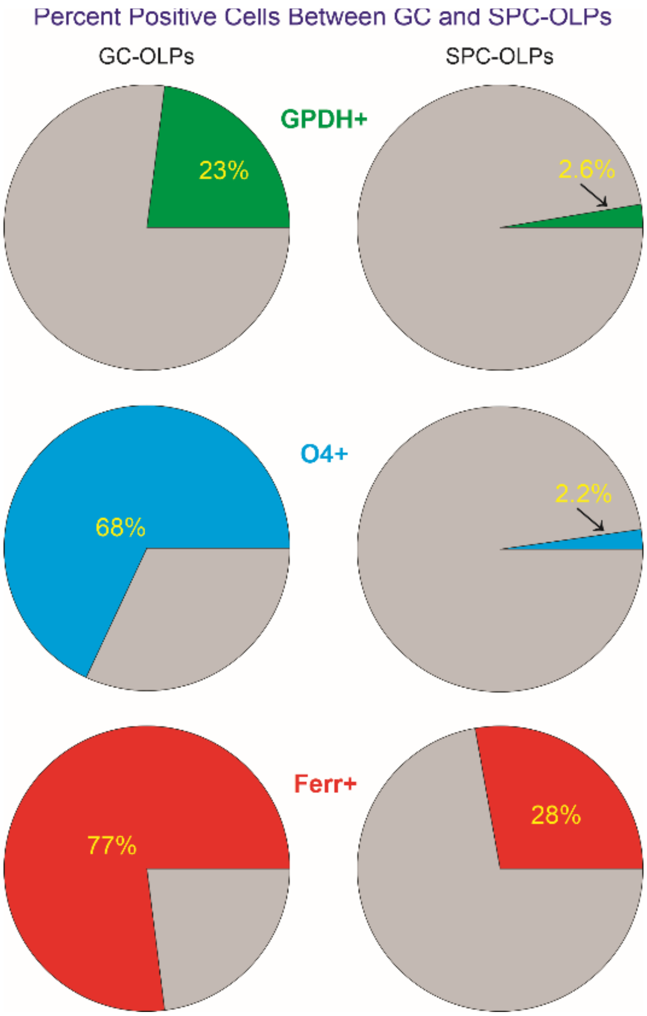

Supplemental Figure S3. View of the expression of OLP markers by GC-OLPs (left panels) and SPC-OLPs (right panels). In GC-OLPs cultures the expression of GPDH was 20% higher than in SPC-OLPs confirming that the GC cells were less immature progenitors than those having been in SPC. The expression of sulfatides detected by the O4 antibody, reached 68% in GC cultures. In contrast, only 2.2% of SPC-OLPs expressed this marker, confirming the degree of immaturity with respect to the GC cells. The iron storing protein ferritin was expressed in 77% of GC-OLPs while only 28% of space flown OLPs expressed this protein.

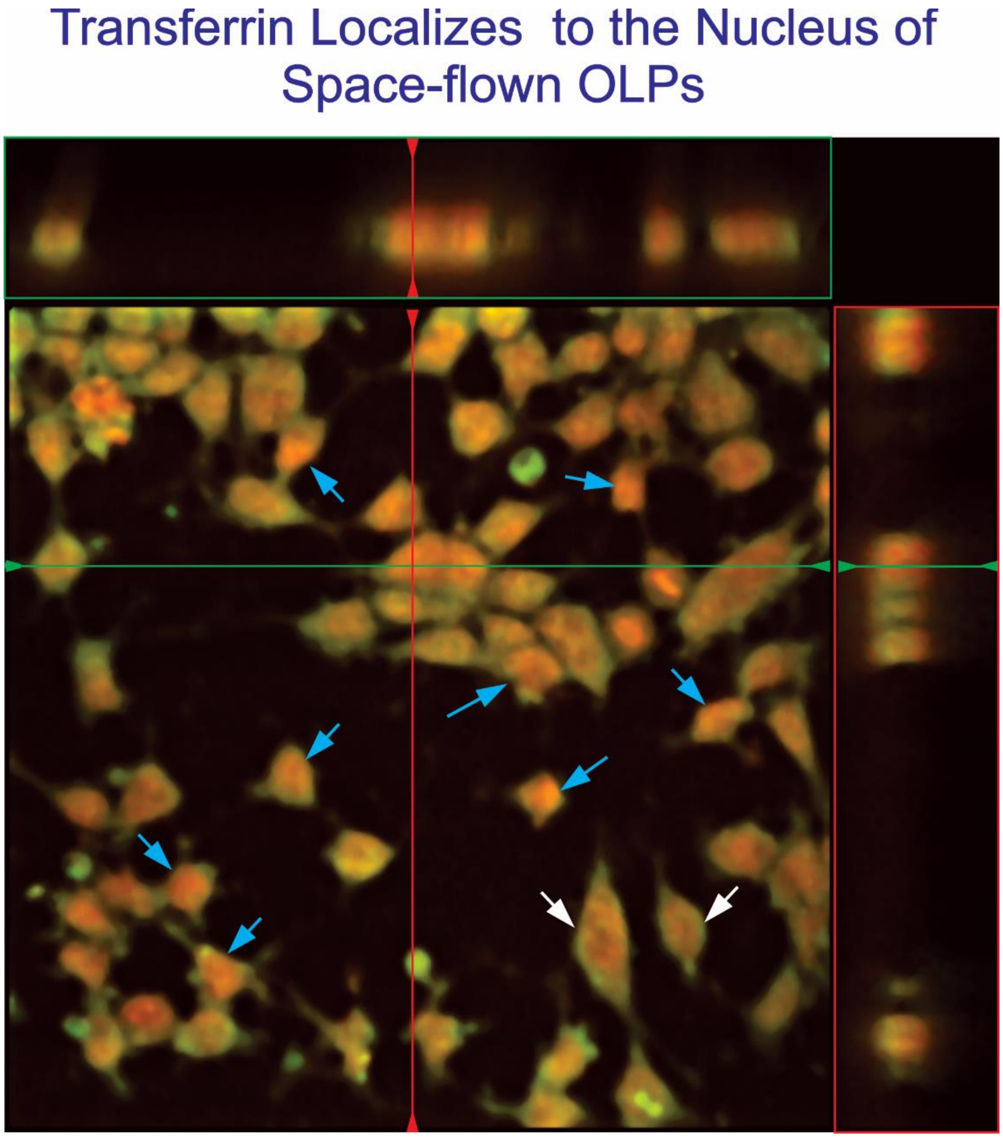

Supplemental Figure S4. Orthogonal view Tf expression to the nucleus of OLPs upon return to Earth. All cells expressed it in the nucleus, examples of them round or with short processes are shown (blue arrows). Bipolar OLPs also expressed it in the nucleus (white arrows).

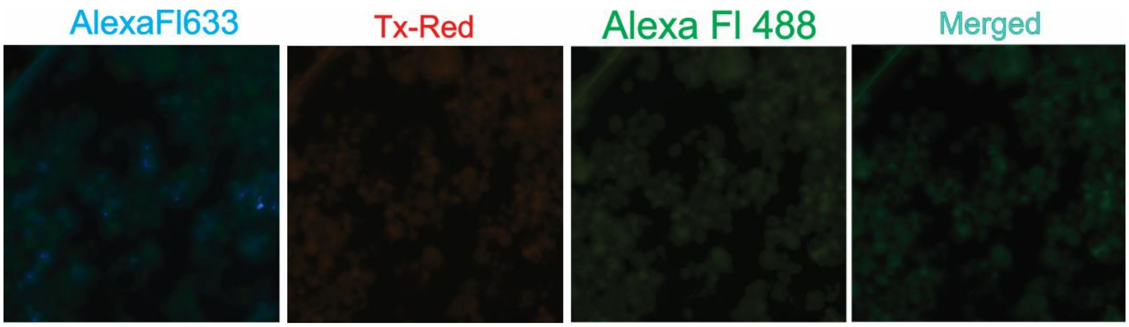

Supplemental Figure S5. Views of the control serum for detection of fluorescent background from the fluorophores used.

Supplemental Table S1. Welcoming culture medium composition.

Reagents	Measurements
Insulin	5 mg
Transferrin	50 mg
Putrescine	16.1 mg
Sodium Bicarbonate	2.2 g
D(+) galactose	4.6 g
Kanamycin	8 µg
IGF-1	88-100ng/ml
*T3	40 ng/ml

*T3 (Triiodothyronine) 20 µg/ml stock solution
